# Large genomic rearrangements within the *PCDH15* gene are a significant cause of USH1F syndrome

**Published:** 2007-01-26

**Authors:** Sandie Le Guédard, Valérie Faugère, Sue Malcolm, Mireille Claustres, Anne-Françoise Roux

**Affiliations:** 1 Laboratoire de Génétique Moléculaire du CHU de Montpellier, Institut Universitaire de Recherche Clinique, Montpellier, France; 2 Clinical and Molecular Genetics, Institute of Child Health, London, United Kingdom

## Abstract

**Purpose:**

Protocadherin-15 (*PCDH15*) is one of the five genes currently identified as being mutated in Usher 1 syndrome and defines Usher syndrome type 1F (USH1F). When *PCDH15* was systematically analyzed for mutations in a cohort of USH1 patients, a number of deletions were found. Here we characterize these deletions as to extent, position, and breakpoints.

**Methods:**

Microsatellite and single nucleotide polymorphism (SNP) analyses, used in a preliminary survey of an Usher cohort of 31 patients, revealed large deletions in three patients. These deletions were further characterized by semiquantitative PCR assays to narrow down the breakpoints.

**Results:**

The analysis of the three large deletions revealed that all six breakpoints are different. The breakpoint junction was identified in one patient and the four other breakpoints were mapped to 4 kb. There were no specific distinguishing features of the isolated breakpoints.

**Conclusions:**

A complete screen of *PCDH15* should include a search for large deletions. Failure to screen for gross genomic rearrangements is likely to significantly lower the mutation detection rate. A likely explanation for the high rate of such deletions is the unusual gene structure. *PCDH15* gene spans nearly 1 Mb for a corresponding open reading frame (ORF) of 7,021 bp. The intron sizes of *PCDH15* are up to 150 kb, and the first three exons of the gene cover 0.42 Mb. The genomic structure of any gene should be taken into consideration when designing a mutation screening strategy.

## Introduction

Usher Syndrome type 1 (USH1) is the most severe form of Usher syndrome [1] and is characterized by congenital profound deafness, vestibular areflexia, and (generally) early onset of retinitis pigmentosa (RP). Six loci have been mapped and five genes have been identified: myosin VIIa (*MYO7A*), cadherin-23 (*CDH23*), protocadherin-15 (*PCDH15*), harmonin (*USH1C*), and SANS (*USH1G*) [2,3]. *MYO7A* appears to be the most frequently involved, and mutations have been reported in 29-54 [4,5] percent of cases but there have been few systematic studies on a cohort of patients [6,7].

The USH1F locus was mapped about ten years ago to chromosome 10q21-22, and the *PCDH15* gene was cloned in 2001 [8]. Several USH1F transcripts have been identified in humans, and the longest isoform (isoform A), consisting of 1 noncoding and 32 protein-coding exons, encodes a 1955 amino acid transmembrane protein that is predicted to contain 11 cadherin repeats, one transmembrane domain, and a cytoplasmic domain containing two proline-rich regions [8-10]. Recently, multiple alternative protocadherin-15 transcripts were characterized in the mouse inner ear. These transcripts define four major isoform classes alternatively spliced, and two of them encode new cytoplasmic domains, raising the number of exons to 39. Three of these isoforms have different spatiotemporal expression patterns in developing and mature hair cells, suggesting a specific role for each protocadherin-15 isoform in the sensory hair bundle [11]. These alternatively spliced exons encoding the two novel cytoplasmic domains were also detected in human retina, indicating that the organization of the human gene could be more complex than was initially thought [11]. Together with other USH1 proteins protocadherin-15 ensures hair bundle morphogenesis [12] via its binding to harmonin [13,14] and myosin VIIa [15].

Around 25 mutations have been documented, nearly all predicted to lead to premature termination of the proteins (6-10). Ouyang et al. [6] studied *PCDH15* together with other USH1 genes in a cohort of patients and found *PCDH15* mutations in five patients but identified both causative mutations in only one of them.

We present in this study an exhaustive analysis of the deletions that were detected in three different families [7]. We show that not only are all deletions different, they also account for a significant proportion of *PCDH15* mutations, probably because of the genomic structure of this gene. We suggest that deletion screening should be part of the molecular analysis for *PCDH15* and any other genes that have such an unusual genomic structure.

## Methods

### Patients

The project was approved by the local ethics committee. Consent to genetic testing was obtained from adult probands or parents of minors. Patients meeting the diagnostic criteria for USH1 were previously described [7]. USH1 was diagnosed on the basis of congenital profound sensorineural deafness, vestibular dysfunction, and retinal degeneration.

U153 and U297 were sporadic cases whereas two affected siblings were available for family U382. All patients underwent audiological examination and all presented with profound deafness. The age of walking was delayed and ranged from 18 months (U153) to 36 months (U297). Electroretinograms (ERG) and fundus examinations were altered in all cases when diagnoses were made at 9 years old (U153 and U297). The ERG was already extinguished at 4 years old in both siblings in family U382.

### Sequencing analysis of *PCDH15*

PCR amplification and sequencing of the *PCDH15* gene, corresponding to isoform A as described by Ahmed et al. [10] (NM_033056), has already been reported [7]. PCR parameters and primers have already been published in the study from Roux et al. [7].

### Haplotypes

**Figure 1:**
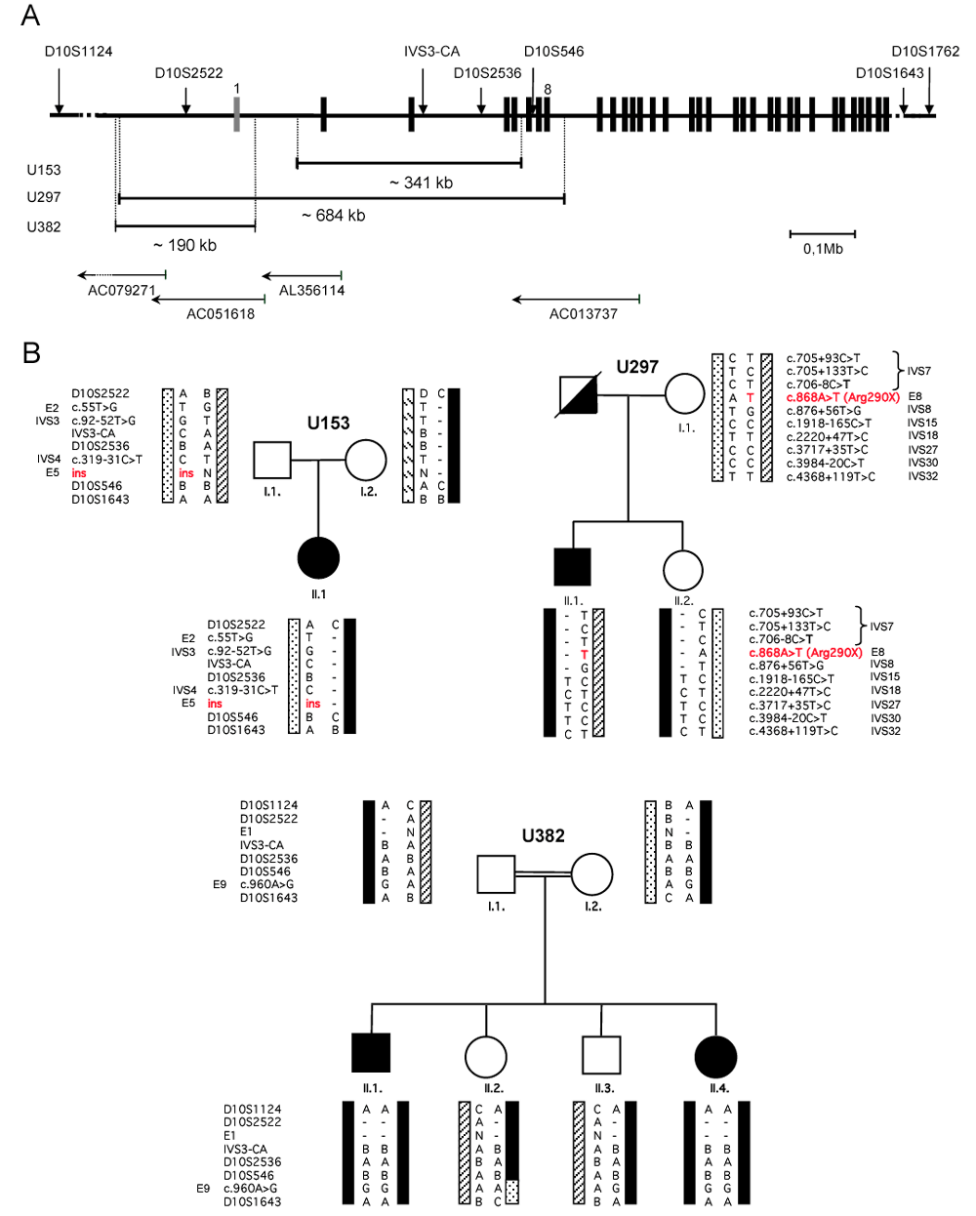
Pedigrees at the USH1F locus. **A**: Genomic organization of the *PCDH15* isoform A with numbering of the exons as described by Ahmed et al. [10] NM_033056. The noncoding exon 1 is represented in grey. Positions of the BAC clones and the microsatellite markers are indicated. **B**: Representation of the three pedigrees with haplotypes. (-) represents lack of amplification; N represents normal. In the first pedigree "Ins" stands for the c.423_430dup mutation (insertion of 7 bp). The different haplotypes are indicated by rectangles with various fillings.

Haplotypes were constructed from a combination of intragenic single nucleotide polymorphisms (SNPs) and seven microsatellite markers: D10S1124-D10S2522-*PCDH15*(IVS3-(CA)-D10S2536-D10S546)-D10S1643- D10S1762. The location of the markers is reported in Figure 1A. Sequences of the microsatellite primers are available on gdb with the exception of IVS3-(CA)-IVS3-F: 5'-GTA TGT ACA GTT AAT TGG TAG-3'; IVS3-R: 5'-GAT GCA GGT ATG GTT TCA G-3'.

Microsatellites were analyzed on an ABI 3100 Avant genetic analyzer (Applied Biosystems, Applera France, France) whereas the SNPs were analyzed by direct sequencing.

### Semiquantitative assays

Two semiquantitative approaches were used in parallel: the quantitative multiplex PCR of short fluorescent fragments (QMPSF) and semiquantitative nonfluorescent multiplex PCR. QMPSF containing multiplex PCR of 3-9 amplicons were analyzed on an ABI310 (Applied Biosystems). We applied to the PCDH15 gene the stategy used by Audrezet et al. for the CFTR gene [16]. Semiquantitative nonfluorescent multiplex PCR products were separated under nondenaturing conditions on a liquid chromatography system (3500 Wave HS system coupled to an HSD system, Transgenomic, Elancourt, France) then quantified by fluorescent detection using a post column intercalation dye, based on guidelines described by Dehainault et al. [17]. One advantage of the semiquantitative nonfluorescent multiplex PCR analyzed on the 3500 Wave HS system is that the primers used for routine sequencing can also be used to determine if a particular exon has been deleted.

**Figure 2:**
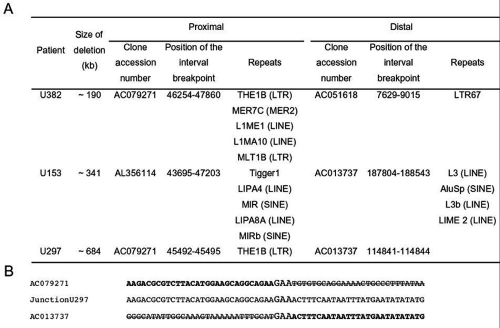
Schematic localization of the deletion breakpoints on the *PCDH15* gene and their analysis. **A**: Localization of the six deletion breakpoints on the bacterial artificial chromosome (BAC) clones. **B**: The breakpoint junction fragment identified in patient U297 is aligned with the wild-type sequences spanning the 5' and 3' breakpoints. The deleted sequences are crossed out.

To narrow down the deletion breakpoints, we used PCR walking methods that included laboratory-designed amplicons localized in a first step every 50 kb both upstream and downstream of the identified deletions. The primers were chosen according to the sequence of the bacterial artificial chromosome (BAC) clones (their accession number is given in Figure 1A). Once a breakpoint was localized between two adjacent amplicons, further primers were designed for new amplicons until this initial 50 kb distance was reduced to a maximum 4 kb interval. Each breakpoint interval thus characterized by PCR walking is positioned on the BAC clones (Figure 2A).

### Identification of the junction fragment in patient U297

Once proximal and distal amplicons were identified within 4 kb intervals, a junction fragment of 1.3 kb was obtained using the forward primer of the proximal amplicon with the reverse primer of the distal amplicon. Further internal primers (U297-bkp-prox-F: 5'-TGA AGA AAC CAC TAA GAC TGA G-3' and U297-bkp-dist-R: 5'-GTA GCC ATT GCA GGC ACA G-3') enabled the sequencing of a 360 bp junction fragment.

### Analysis of control DNA

Guthrie cards were obtained from the neonatal screening center GREPAM in Montpellier. All samples were anonymously referenced and neither phenotypic nor ethnic origin data were available. DNA was extracted using standard procedures. A total of 172 control DNAs were amplified for the noncoding exon 1 and exon 2. We tested 88 DNAs using primers U297-bkp-prox-F and U297-bkp-dist-R.

## Results

### Haplotype analyses and evidence of the deletions

We have previously reported the screening for mutations in *MYO7A*, *CDH23*, *PCDH15, USH1C*, and *SANS* in a cohort of 31 USH1 families [7]. While conducting a preliminary linkage analysis using microsatellites surrounding each USH1 gene, we detected apparent noninheritance of some markers or failure of amplification among the USH1F panel (D10S1124; D10S2522; IVS3-(CA); D10S2536; D10S546; D10S1643 and D10S1762) in two families (U153, and U382) as shown on Figure 1B. These results were confirmed by amplification in neighboring regions. Microsatellite analysis was not informative in patient U297 but a deletion was suggested after an apparently homozygous p.Arg290X mutation, localized in exon 8, was identified. This novel mutation appeared to be carried on different haplotypes as revealed by heterozygous intronic SNPs in the 3' end of the gene (Figure 1B).

The resulting haplotypes of the three pedigrees are presented in Figure 1B together with SNPs analyses when informative. Sequencing of the entire coding region of *PCDH15* revealed that the patients were compound heterozygotes for premature truncating mutations p.Ser144LeufsX15 (c.423_430dup) and p.Arg290X (c.868A>T) in trans to the two deletions identified in U153 and U297. A third homozygous deletion was identified in family U382 with a known history of consanguinity.

### Narrowing of the breakpoints

To narrow down the deletion breakpoints of the two compound heterozygous patients, we used two semiquantitative PCR walking methods. The narrowing of the deletion breakpoint in patient U382 was performed by simple PCR walking, looking at the amplification or nonamplification of each amplicon.

When each breakpoint was localized within an interval below 4 kb, PCR was performed to identify the precise deletion breakpoints. One breakpoint was identified (U297; see Figure 2B). Unfortunately, several other attempts using different long-range PCR kits failed to identify a junction fragment in the other two patients, suggesting that the deletions may be more complex than anticipated.

PCR walking in patient U382 narrowed the deletion to a proximal breakpoint localized within a 1.6 kb interval in the 5' region of the *PCDH15* gene and to a distal breakpoint lying in IVS1 within an 1.4 kb interval. The size of the deletion is estimated as 190 kb (Figure 1 and Figure 2). None of the 172 control DNAs showed an absence of amplification of exon 1 excluding a similar homozygous deletion in these controls.

The deletion in patient U153 was originally characterized as spanning exons 3-5 by means of the intragenic marker D10S2536 and SNP. However this deletion was further characterized as extending from a proximal breakpoint within IVS1, within an interval of 3.5 kb, to a distal breakpoint within an 0.8 kb interval of IVS5 (Figure 1 and Figure 2). The size of the deletion is about 341 kb. None of the 172 control DNAs showed an absence of amplification of exon 2, excluding an homozygous deletion spanning at least this exon in these controls.

The deletion in patient U297 was narrowed down by semiquantitative PCR then further characterized by amplification of a junction fragment (Figure 2B). The deletion spans about 684 kb, includes exon 8, and is in trans to the p.Arg290X mutation (Figure 1 and Figure 2). The proximal breakpoint, lies within 2.4 kb of U382 breakpoint, but is not identical. This junction fragment was not detected in 176 alleles (i.e., 88 control DNAs).

## Discussion

None of the deletions described in this paper have been found in normal controls, and they would all result in nonfunctional protein. Recent data has shown that multiple isoforms exist. No exon is present in all identified so far alternative transcripts [11]. However, no isoform has been observed to containing deletions extending from exon 2 to exon 5 or 8. In addition, an Arg3X mutation in the second exon has been observed in two patients, providing evidence that the presence of the first few exons is necessary [8,9]. Recently, an alternatively spliced isoform (lacking exons 3-15) was found to circumvent the effect of the mutant allele IVS14-2A>G in the homozygous *Pcdh^15av-5J^* mice [18]. Such a mechanism is not likely to be involved here as the patients described in this study are affected with typical USH1.

The six intervals surrounding the breakpoints were placed on the BAC clones (NCBI Accession numbers given in Figure 1A and Figure 2A) and analyzed through repeat masker (repeatmasker). Although short interspersed elements (SINE), long interspersed elements (LINE), and long terminal repeats (LTR) were found in all cases (Figure 2A), there is no evidence for direct repeats or duplicons as found in some other cases of recurrent deletion or duplication. In patient U297 the only obvious feature at the breakpoint is a repetition of GAA (Figure 2B). This is in line with the findings in studies of deletions of the dystrophin gene in DMD [19] and duplications of *PLP1* in Pelizaeus-Merzbacher disease [20].

A detailed analysis of the gene structure provides a likely explanation for the high rate of such deletions. The gene spans nearly 1 Mb for a corresponding ORF of 7,021 bp. The intron size of *PCDH15* is up to 150 kb, and the first three exons of the gene cover 0.42 Mb. The six breakpoint intervals lie in introns ranging from 22 to 140 kb in size localized in the first third of the gene.

Because of the large size of *PCDH15* and, in particular, the low proportion which is coding, predominantly in the 5' end of the gene, it is not surprising that large deletions, with differing breakpoints, form a significant proportion of *PCDH15* mutations (30% in our cohort) which represents nearly 10% of all USH1 patients [7]. The situation is reminiscent of the high frequency of deletions within the dystrophin gene found in patients with Duchenne Muscular Dystrophy. The dystrophin gene coding region of 11 kb is encoded over 2.4 Mb of genomic DNA. Around 60% of mutations are large deletions [21], and many occur within the two large introns 7 and 44 [22].

This observation has several implications. First, although an initial linkage analysis approach was incorporated to target which gene was the best candidate for mutation screening, it may also identify apparent noninheritance of markers. Second, these results show that restricting the molecular analysis of *PCDH15* in USH1F to sequencing is not sufficient, and testing for large genomic rearrangements is recommended. A previous study has described only one *PCDH15* mutation in patients [6]. It is possible that either a large genomic deletion or a mutation lying in the additional exons [11] accounts for the second pathogenic mutation in these patients. Detection of large genomic rearrangements is becoming easier and more routine with the development of methods such as multiplex ligation-dependent probe amplification (MLPA) and multiplex amplifiable probe hybridization (MAPH) [23] and should be considered, particularly for genes with an extended genomic structure. Third, large genomic rearrangement analysis cannot be routinely achieved by PCR of junction fragments as each deletion appears to be different and is likely to be ineffective across breakpoints involving complex rearrangements. PCR may still be customized by semiquantitative PCR, or other methods, such as MLPA, may be developed.
